# The Effect of Chronic Methamphetamine Treatment on Schizophrenia Endophenotypes in Heterozygous Reelin Mice: Implications for Schizophrenia

**DOI:** 10.3390/biom10060940

**Published:** 2020-06-22

**Authors:** Camilla Hume, Shelley Massey, Maarten van den Buuse

**Affiliations:** School of Psychology and Public Health, La Trobe University, Melbourne, VIC 3086, Australia; c.hume@latrobe.edu.au (C.H.); shelley.massey.8@gmail.com (S.M.)

**Keywords:** reelin, psychosis, methamphetamine, sensitization, dopamine, social behaviour, prepulse inhibition

## Abstract

Reelin has been implicated in the development of schizophrenia but the mechanisms involved in this interaction remain unclear. Chronic methamphetamine (Meth) use may cause dopaminergic sensitisation and psychosis and has been proposed to affect brain dopamine systems similarly to changes seen in schizophrenia. We compared the long-term effect of chronic Meth treatment between heterozygous reelin mice (HRM) and wildtype controls (WT) with the aim of better understanding the role of reelin in schizophrenia. Meth pretreatment induced sensitisation to the effect of an acute Meth challenge on locomotor activity, but it had no effect on baseline PPI or sociability and social preference. In all behavioural models, HRM did not significantly differ from WT at baseline, except spontaneous exploratory locomotor activity which was higher in HRM than WT, and sociability which was enhanced in HRM. Locomotor hyperactivity sensitisation was not significantly different between HRM and WT. Chronic Meth treatment reduced spontaneous locomotor activity to the level of WT. No deficits in PPI or social behaviour were induced by chronic Meth pretreatment in either strain. In conclusion, these data do not support a role of reelin in schizophrenia, at least not in HRM and in the methamphetamine sensitisation model.

## 1. Introduction

Reelin is an important extracellular protein involved in neuronal migration and brain layer formation during prenatal neurodevelopment. In adulthood it plays a complex role in synaptic plasticity by altering dendritic spine morphology, regulating N-methyl-D-aspartate (NMDA) glutamate receptor function and influencing neurotransmitter synthesis [1,2]. Reelin is secreted by selected GABA interneurons and binds to specific receptors (ApoER 2 and VLDL-R) to trigger signalling cascades [2]. Post-mortem studies of individuals with schizophrenia and bipolar disorder with psychosis have shown a 50% down-regulation of reelin within the brain [3]. Conversely, reelin supplementation in the form of a single ventricular injection has been shown to recover schizophrenia-like deficits in mice [4]. These findings have led to the suggestion that this protein may play an important role in schizophrenia vulnerability [1,3,5]. However, it remains unclear through which neurotransmitter mechanisms reelin plays a role in schizophrenia.

The heterozygous reelin mouse (HRM) has been widely used to study the neuroanatomical and behavioural effects of reelin deficiency. The HRM carries one mutated reelin gene (RELN) allele and one normal RELN allele leading to a ≈50% reduction in reelin protein levels in the brain, similar to the deficit observed in schizophrenia [3,6]. This is associated with neuroanatomical changes that are also seen in human populations with schizophrenia, such as decreased neuropil expression and reduced dendritic spine density [7,8]. Additionally, some studies suggest that the HRM has subtle behavioural characteristics related to schizophrenia [9,10].

Altered functioning of the dopamine pathways in the brain is associated with the positive symptoms seen in schizophrenia [11,12]. The mesolimbic and nigrostriatal pathways projects from the ventral tegmental area (VTA) and substantia nigra to the ventral and dorsal striatum, respectively. DA receptor agonists and psychostimulants like methamphetamine (Meth) increase dopaminergic transmission within these systems. In contrast, DA receptor antagonists and antipsychotics reduce positive symptoms, although they are suggested to have little effect on negative and cognitive symptoms of schizophrenia [13]. Brain imaging studies have shown that brain dopamine transmission in schizophrenia is increased [14,15,16]. For example, while baseline dopamine release was not altered, an acute amphetamine challenge evoked a significantly greater increase of dopamine release, as measured by reduced dopamine D2 receptor availability, in the striatum of patients with schizophrenia compared to healthy controls [14]. Findings like this have led to the suggestion that dopamine transmission in schizophrenia is in a state of endogenous sensitization [17]. Of note, studies have shown that HRM show a selective decrease in markers of DA neurons in the midbrain [18], increased expression of dopamine D2 receptors in the forebrain [19], and reduced levels of dopamine in the frontal cortex [20]. However, striatum dopamine levels [20] and binding density of dopamine D2 receptors and dopamine transporters [21] were not different between HRM and WT controls. No previous studies have addressed dopaminergic sensitisation in these animals.

Meth is an amphetamine derivative that distributes rapidly in the central nervous system due to its highly lipophilic properties [22]. This results in rapid onset of CNS effects such as euphoria, alertness, elevated self-esteem and excessive sympathetic activation [23]. Meth principally increases dopamine release but also norepinephrine and serotonin (5-HT) [24]. Acute Meth exposure causes the release of stored dopamine by acting on the dopamine transporter (DAT) and vesicular monoamine transporter-2 (VMAT2), leading to increased dopamine concentrations in the synapse [24,25]. Chronic Meth exposure leads to neuroadaptive changes that result in behavioural sensitization and potential psychosis. In this context, behavioural sensitization is defined as the progressive amplification of behavioural responses to a drug after repeated administration that persists despite periods of abstinence [25]. Due to a range of neurological and behavioural similarities, Meth is suggested to alter similar neurochemical pathways that are also implicated in schizophrenia [23]. Indeed, in a recent study, repeated oral amphetamine amplified forebrain dopamine release in health volunteers to the level seen in first-episode psychosis patients [26]. The effects of chronic Meth may therefore be a useful model to investigate sensitisation mechanisms in schizophrenia.

Meth psychosis may be prolonged and relapse may occur spontaneously despite abstinence; in contrast, not all individuals who use Meth go on to develop psychosis [27,28,29]. Meth users with first-degree relatives predisposed towards schizophrenia are more likely to experience psychotic symptoms than users with no family history [30]. This suggests that as well as neuroadaptive changes, genetic and developmental vulnerabilities are also implicated in the development of psychosis and schizophrenia. Given its role in neurodevelopment and postulated role in schizophrenia, reelin may be one such factor.

The aim of the present study therefore was to investigate the role of reelin in schizophrenia by using a chronic Meth treatment paradigm [31,32] and assessing its effect on a number of schizophrenia-relevant endophenotypes in adulthood, including locomotor activity (both spontaneous and acute Meth induced), prepulse inhibition (PPI) and social interaction [33,34,35]. It was hypothesised that HRM would be more vulnerable to a three-week Meth pretreatment than WT mice, and would develop more severe behavioural characteristics associated with psychosis. Compared to saline-pretreated mice, Meth-pretreated mice were expected to show sensitization, reflected as greater locomotor hyperactivity when given an acute Meth challenge. It was also hypothesised that Meth pretreatment would interact with reelin deficiency to induce greater hyperlocomotion than either Meth pretreatment or reelin deficiency alone. PPI has been reported to be disrupted after amphetamine sensitisation similarly to schizophrenia [36]. Deficits in PPI were expected for MA-pretreated mice and Meth-pretreated HRM were expected to show greater PPI deficits than all other experimental groups. Finally, it was expected that Meth-pretreated HRM, but not saline-pretreated HRM, would show less sociability and social preference than all other experimental groups.

## 2. Materials and Methods 

### 2.1. Animals

**Figure 1:**
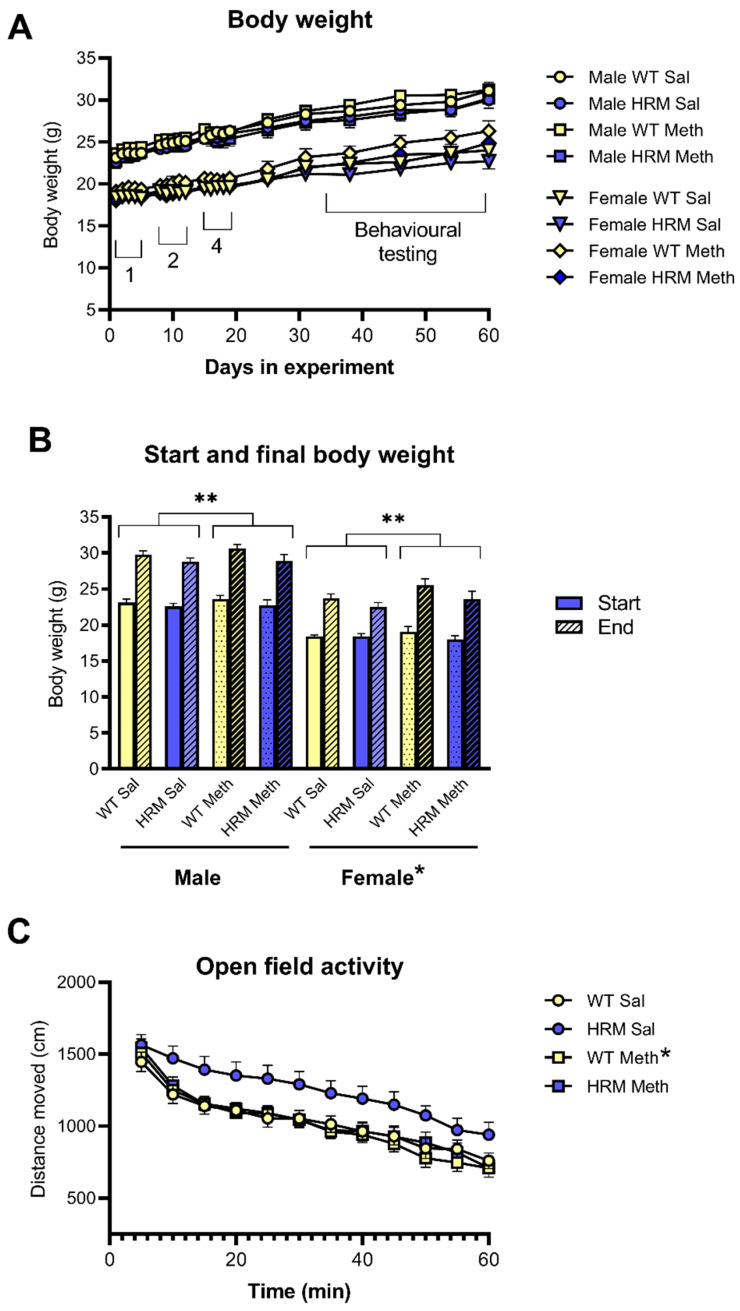
Body weight and spontaneous exploratory locomotor activity of male and female WT and HRM treated chronically with saline or Meth. **Panel A** shows body weights during the entire experiment, including treatment with 1 mg/kg (1), 2 mg/kg (2) and 4 mg/kg (4) starting from 6 weeks of age. Behavioural testing was done starting two weeks after the last injection. **Panel B** shows start and end body weight of all groups. * *p* < 0.05 for lower body weights in female mice compared to male mice. ** *p* < 0.05 for lower body weight gain in HRM compared to WT independent of Meth treatment or Sex. **Panel C** shows spontaneous exploratory locomotor activity expressed as distance moved every 5 min before injection of challenge saline or Meth. Data were combined for male and female groups of WT and HRM which were pretreated with saline or Meth. * *p* < 0.05 for higher activity in HRM-pretreated with saline compared to the other groups. Exploratory activity habituated over the one-hour period but there were no other differences between the groups.

All mice were obtained from a breeding colony [6,21] at the Central Animal House (CAH) of La Trobe University, Melbourne. Genotyping was done by Transnetyx (Cordova, TN, USA) using tissue obtained while ear-tagging the mice at weaning. HRM and WT C57Bl/6 littermates were used and experimenters were blind to genotype. Several overlapping cohorts of mice were used with varying genotype and sex ratios, depending on breeding success. Group size was 11–19 and all mice were tested in all three behavioural paradigms, except if there were technical data such as equipment failure or data loss (see Table 1 for number of mice per genotype/behavioural test). Mice were group-housed with two to five sex-matched animals per cage, and kept on a 12-h light/dark cycle (lights on at 7 am). Housing consisted of individually-ventilated cages (IVC, Tecniplast, Buguggiate, Italy) with enrichment in the form of corn cob bedding, paper furl and tunnels. Room temperature remained at 21 ± 2 °C and mice had ad libitum access to pellet food and water. Behavioural testing, injections and handling took place during the light cycle between 8 am and 5 pm. All procedures were approved by La Trobe University’s Animal Ethics Committee in accordance with the Australian Code of Practice for the Care and Use of Animals for Scientific Purposes set out by the National Health and Medical Research Council of Australia (NHMRC). Health status and body weight of the animals was recorded regularly (Figure 1).

### 2.2. Methamphetamine Treatment

Methamphetamine ((±)-methamphetamine hydrochloride, Meth) was purchased from the National Measurement Institute, Canberra, Australia, and dissolved in sterile saline (0.9% NaCl). Saline vehicle control solution was used for all injections. Cages of mice were randomly allocated to Meth or saline pretreatment and the mice were treated for three weeks beginning at six weeks of age as previously described [31,37]. Briefly, in order for mice to replicate tolerance effects seen in MA users [27], an increasing dose of Meth was administered each week [31,37]. Week 1 involved once-daily intraperitoneal (IP) injections of 1 mg/kg of Meth or saline solution, Monday to Friday. Week 2 involved twice-daily IP injections of 2 mg/kg of Meth or saline, morning and afternoon, Monday to Friday. Week 3 involved twice-daily IP injections of 4 mg/kg of Meth or saline, morning and afternoon, Monday to Friday. Injection volume was 5 mL/kg [31,37]. Following the last treatment, the mice were left undisturbed for at least two weeks.

### 2.3. Behavioural Testing

Starting at 11 weeks of age, each mouse was consecutively tested for changes in social interaction, baseline prepulse inhibition (PPI) and for locomotor activity sensitization to an acute challenge dose of 1 mg/kg or 3 mg/kg of Meth. There were 3–4 days between behavioural tests.

#### 2.3.1. Social Interaction and Social Preference

Social interaction was tested using a three-chamber plexiglass apparatus as previously described [32,33,38]. Briefly, the box measured 64 × 30 cm and was divided into three chambers (21 × 10 cm) with entrances to allow the subject mouse to move between chambers. Younger mice (approximately 6–8 weeks old) from the same colony were used as ‘stranger’ mice and were used in multiple trials. These stranger mice were placed in small wire mesh enclosures in either of the outer chambers, with the location alternated between trials to control for any chamber bias of test mice.

The subject mouse was initially placed in the centre chamber for five minutes, and subsequently was allowed to explore all empty chambers for another five minutes. After this total of ten minutes, a stranger mouse, was placed into one of the outer chambers in its mesh enclosure. An empty enclosure was placed in the opposite chamber. In the sociability phase of the experiment the subject mouse was allowed explored the entire arena for ten minutes. In the social preference phase of the experiment, a novel stranger mouse was placed in the previously empty chamber and the subject mouse was again allowed to explore all chambers for ten minutes. Between sessions, the arena and cages were cleaned thoroughly with 10% ethanol solution and non-scented soapy water.

Video recordings of movement, chamber visits and sociability were analysed off-line using Ethovision software (Noldus, Wageningen, The Netherlands). Time spent in each chamber and time spent with the nose-point in close proximity to the enclosures was assessed. Sociability was defined as the amount of time the subject mouse spent investigating the stranger mouse compared to the empty cage. Social novelty preference was defined as the amount of time the subject mouse spent investigating the novel stranger mouse vs. the now familiar mouse. As it is a more reliable indicator of social interaction, only time spent within the sniffing perimeters will be presented here.

#### 2.3.2. Prepulse Inhibition of Acoustic Startle (PPI)

All mice were tested for PPI and startle amplitudes using six sound-attenuating startle chambers with a plexiglass cylinder and motion sensor in each (SR-Lab; San Diego Instruments, San Diego, CA, USA; see [21,39] for details). Testing consisted of 104 trials and was concluded in approximately 36 minutes. Background noise was set at 70 dB and 8 trials used no stimulus to detect excessive spontaneous movement. Testing started and ended with a block of 8 pulse-alone trials involving a 40 ms burst of 115dB white noise. The remaining trials delivered a 115 dB startle pulse preceded either 30 ms or 100 ms by a prepulse (PP) set at 2, 4, 8, or 16 dB above background noise (72, 73, 78 and 86 dB). Inhibition of startle was calculated as the difference between response to prepulse-pulse trials and pulse-alone trials, analysed as the percentage of response to pulse-alone trials. Animals underwent a pre-test three days prior to experimental testing to habituate them to the testing environment which involved running the same protocol as described above.

#### 2.3.3. Locomotor Activity and Meth-Induced Locomotor Hyperactivity

Each mouse underwent three individual locomotor activity tests on three separate days. Mice were placed in individual open field locomotor chambers (28 L × 28 W × 19 H cm) (Med Associates Inc. St. Albans, VT, USA), containing rows of 24 photocell beam monitors located 1 cm apart. Locomotor distance moved was calculated every 5 min from the number of beam breaks. The middle 8 photobeams were used to calculate inner zone activity as a measure of anxiety (Supplementary Table S1). Each mouse was first habituated to the arena for 60 min prior to receiving an IP injection, and then locomotor activity was recorded for another 120 min. Locomotor activity sessions consisted of administration of saline (0.9% NaCl) during test 1, a low-dose Meth challenge (1 mg/kg) during test 2 and a high-dose Meth challenge (3 mg/kg) during test 3. The order of injection 

Dose was not randomised to avoid carry-over effects of the high-dose Meth challenge. Two or three days were left between tests. Data from the three pre-injection habituation phases were averaged to obtain a measure of spontaneous exploratory locomotor activity (Figure 1).

### 2.4. Data Analysis

All statistical analysis was conducted using IBM SPSS Statistics Version 22 (IBM Australia, St. Leonards, Australia). Data were checked for outliers and violations of normal distribution by examining normal Q-Q and box plots and using Kolmogorov-Smirnov’s Test of Normality. Homogeneity of variance was checked using Levene’s test for Equality of Variance. Sphericity was assessed using Mauchly’s Test of Sphericity, and as this assumption was often violated the more conservative Greenhouse-Geisser corrected *p* value was used for all analyses. Mixed-between-within-subjects analyses of variance (ANOVAs) were used to analyse locomotor activity, PPI, and social interaction data. Between-subjects factors were drug pretreatment (saline, Meth), reelin genotype (WT, HRM) and sex of the animals (male, female). For locomotor activity analysis, the additional within-subjects factor was acute treatment dose (saline, 1 mg/kg Meth, and 3 mg/kg Meth challenge). For PPI analysis, an additional within-subjects factor was prepulse (PP) intensity levels (2, 4, 8, 16 dB above background noise). For social interaction analysis, the within-subject factor was time spent sniffing in the social versus control perimeter during the sociability phase, and time spent in the social versus familiar perimeter during the social preference phase. Other than for body weight, behavioural data expressed in the figures did not show relevant sex differences is therefore for males and females combined. Additional analysis (Supplementary Table S1) revealed some sex differences but this never interacted statistically with either Genotype or Meth treatment. In all cases, when *p* < 0.05, differences were considered statistically significant.

## 3. Results

### 3.1. Body Weight

Analysis of all body weight data (Figure 1A) showed the expected increase over the course of the experiment (main effect of Time: F(3.47,298.6) = 468.6, *p* < 0.001) and generally higher body weights in male mice than females (main effect of Sex: F(1,86) = 223.1, *p* < 0.001; Time x Sex interaction: F(3.47, 298.6, *p* < 0.001). HRM had slightly lower body weight gain than WT (Time x Genotype interaction: F(3.47, 298.6) = 4.16, *p* = 0.004) but Meth-treated mice showed greater body weight increase during the experiment than saline-pretreated mice, irrespective of genotype (Time × Meth interaction: F(3.47, 298.6) = 2.73, *p* = 0.036). 

Analysis of body weights at the start and the end of the experiment largely confirmed these trends (Figure 1B; main effect of Time: F(1,86) = 1008.2, *p* < 0.001; main effect of Sex: F(1,86) = 163.0, *p* < 0.001; Time × Sex interaction: F(1,86) = 10.2, *p* = 0.002; Time x Genotype interaction: F(1,86) = 5.56, *p* = 0.021), although the effect of Meth on body weight gain failed to reach significance (*p* = 0.055). At the start of the experiment, other than female mice having lower body weight than male mice (F(1,86) = 160.4, *p* < 0.001), there were no differences between the genotypes or the treatment groups. At the end of the experiment, in addition to the effect of Sex (F(1,86) = 123.6, *p* < 0.001), HRM showed lower body weights than WT, independent of the sex of the animals (F(1,86) = 8.11, *p* = 0.005). Although body weights in Meth-pretreated mice tended to be higher than those in saline controls, this difference did not reach statistical significance (*p* = 0.065). These data show that Meth pretreatment did not result in reduced body weight gain although HRM gained weight slightly slower than WT mice. Although female mice were smaller than male mice, the effects of genotype and Meth were independent of the sex of the animals.

### 3.2. Spontaneous Locomotor Activity in WT and HRM

Spontaneous exploratory locomotor hyperactivity of the mice, before injection of acute challenge treatments, habituated over time (F(4.72, 443.6) = 200.5, *p* < 0.001) but this habituation did not interact with other factors. Locomotor activity was higher in HRM controls compared to the other groups (Figure 1C, main effect of Meth pretreatment and of Genotype, F(1,94) = 4.10, *p* = 0.046, and 4.51, *p* = 0.036, respectively). 

Further analysis of data from saline-pretreated groups showed that spontaneous activity was higher in HRM than in WT mice (F(1,42) = 4.96, *p* = 0.031). Further analysis of data from Meth-pretreated groups showed no genotype difference (Figure 1). Analysis of data from WT mice revealed no effect of Meth pretreatment. In contrast, analysis of data from HRM revealed lower exploratory activity following Meth treatment compared to saline pretreatment (F(1,43) = 4.78, *p* = 0.034). Thus, HRM are hyperactive compared to WT controls, but Meth pretreatment reduces exploratory activity in HRM to the level of WT, where Meth had no effect (Figure 1).

### 3.3. Locomotor Hyperactivity Induced by Acute Meth Treatment in WT and HRM

**Figure 2:**
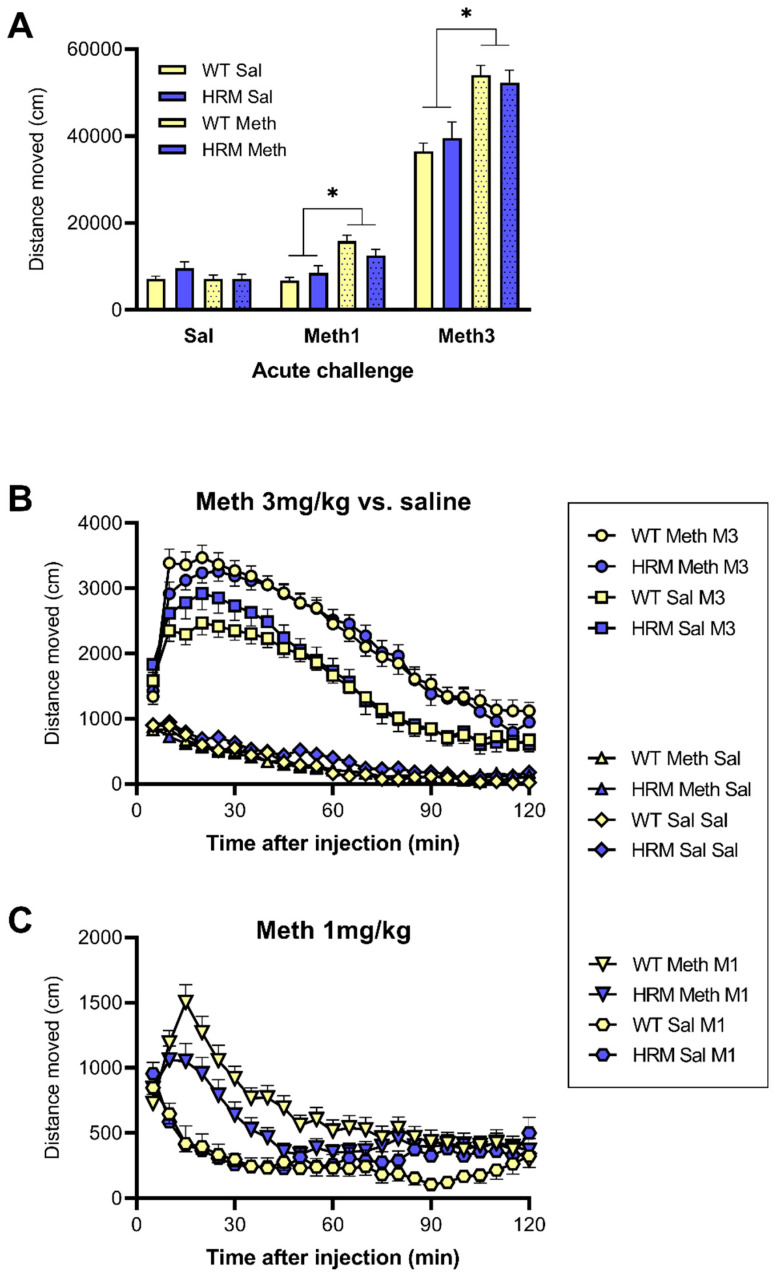
The effect of acute challenge with saline, 1 mg/kg of Meth (Meth1) or 3 mg/kg of Meth (Meth3) on locomotor activity, expressed as distance moved. Panel **A** shows total distance moved over the 2 h post-injection period. * *p* < 0.05 for difference between Meth-pretreated and saline-pretreated mice independent of genotype. Panel **B** shows locomotor hyperactivity every 5 min following acute injection of 3 mg/kg of Meth or saline. Meth-pretreated mice shows significantly greater Meth-induced hyperactivity than saline-pretreated mice but there were no significant genotype effects. There were no differences between the groups following acute saline treatment. Panel **C** shows locomotor hyperactivity every 5 min following injection of 1 mg/kg of Meth. Meth-pretreated mice show significantly greater Meth-induced hyperactivity compared to saline-pretreated mice but the were no differences between the genotypes (see text for details of statistical analysis. Data are mean ± SEM of males and females combined.

As expected, acute treatment with methamphetamine dose-dependently caused locomotor hyperactivity and this effect was enhanced by chronic Meth pretreatment, reflecting sensitization (Figure 2). Analysis of total distance moved over the two hours post-injection (Figure 2A) revealed a main effect of acute Meth dose (F(1.3, 119.9) = 756.3, *p* < 0.001), a main effect of Meth pretreatment (F(1,94) = 24.4, *p* < 0.001) and an interaction between the acute Meth dose and Meth pretreatment (F(1.3, 119.9) = 29.9, *p* < 0.001). However, there was no interaction with genotype, suggesting that these effects were not different between WT and HRM. Further comparison between the groups after each acute treatment confirmed this finding. Thus, following acute saline injection there were no genotype or pretreatment effects; following acute injection of 1 mg/kg or 3 mg/kg there was a significant effect of pretreatment (F(1,94) = 21.2 and 31.9, respectively, *p* < 0.001) but no effect of genotype, reflecting similarly enhanced locomotor hyperactivity following pretreatment with Meth in WT and HRM (Figure 2A, see also Supplementary Table S1).

Detailed analysis of the time course of locomotor hyperactivity following acute Meth treatment confirmed the observations for total two-hour distance moved (Figure 2B,C). Comparison of the effect of the acute 3 mg/kg dose of Meth with saline showed both a main effect of acute (F(1,94) = 838.6, *p* < 0.001) and chronic Meth (main effect F(1,94) = 19.6, *p* < 0.001) and an interaction between these factors (F(1,94) = 40.8, *p* < 0.001), confirming significant sensitization of the effect of this high dose of Meth. There were no other relevant statistical interactions, suggesting there was no difference in this sensitization between WT and HRM (Figure 2B).

Comparison of the effect of the acute 1mg/kg dose of Meth with saline showed both a main effect of acute (F(194) = 34.1, *p* < 0.001) and chronic Meth (F(1,94) = 5.44, *p* = 0.022) and an interaction between these factors (F(1,94) = 51.3, *p* < 0.001), confirming significant sensitization of the effect also at this low challenge dose of Meth (Figure 2C). However, despite HRM showing slightly lower distance moved than WT, again there were no statistical interactions, suggesting sensitisation was independent of genotype (Figure 2C). Inspection of the data (Figure 2B,C) suggested subtle differences between the groups in the first 45 min following acute Meth injection. However, separate analysis of data from the first 45 min showed no significant genotype differences at either the 3 mg/kg or 1 mg/kg Meth challenge dose (data not shown).

### 3.4. Effect of Meth Pretreatment on PPI in WT and HRM

**Figure 3:**
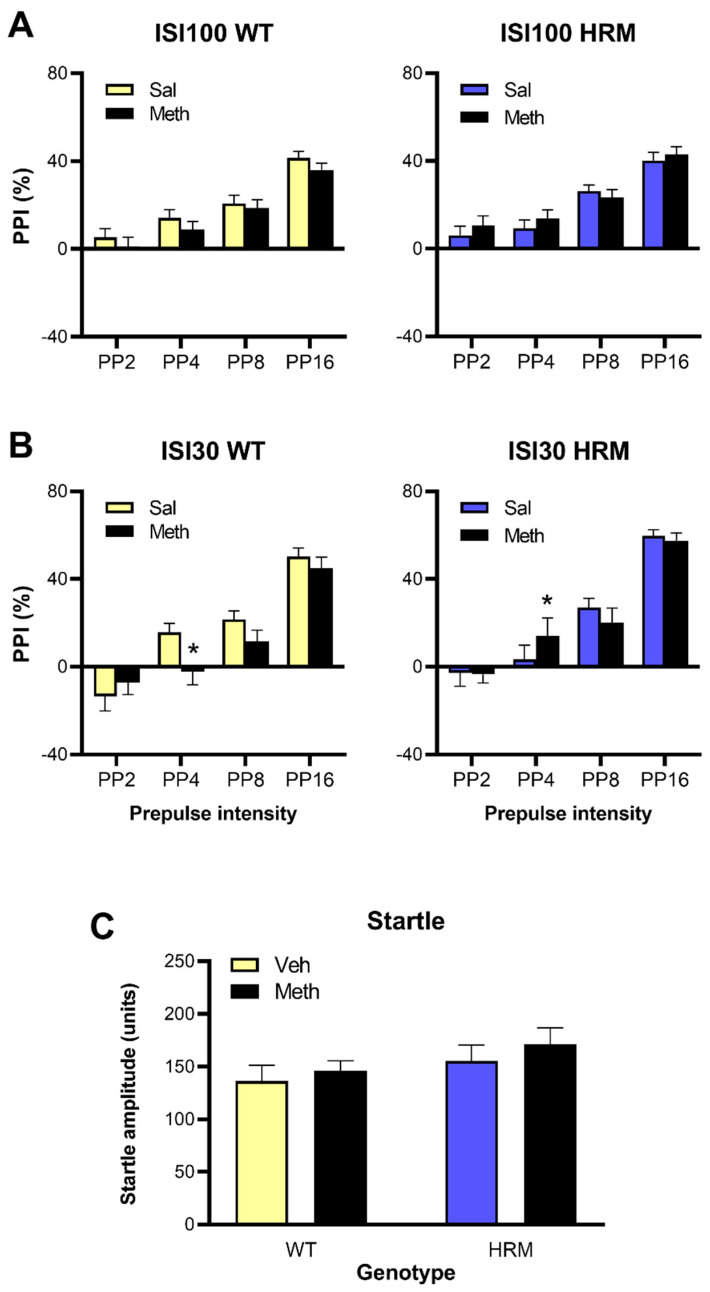
Prepulse Inhibition (PPI) of acoustic startle in WT and HRM pretreated with saline or Meth. Data are expressed as %PPI at prepulse (PP) levels of 2, 4, 8 and 16 dB over baseline (PP2, PP4, PP8, PP16, respectively) and 100 (panel **A**) or 30 ms (panel **B**) inter-stimulus intervals (ISI100 and ISI30, respectively). There were no differences between Meth and saline-pretreated mice except a decrease of PPI at PP4 and ISI30 in WT as opposed to an increase in HRM (* *p* < 0.05). Panel **C** shows there was no difference in average startle amplitudes between the groups. Data are mean ± SEM of males and females combined.

As expected, increasing levels of prepulses caused progressively greater inhibition of startle responses and increased %PPI (Figure 3). There were main effects of prepulse intensity at both the 100 ms and 30 ms ISI (F(3,288) = 130.5 and 148.2, respectively, *p* < 0.001). However, at the 100 ms ISI there were no main effects of genotype or Meth pretreatment and no interactions. At the 30 ms ISI, a Meth × Genotype × Prepulse Intensity interaction (F(3,288) = 3.38, *p* = 0.019) suggested differences in the effect of Meth between the genotypes depending on the prepulse intensity. Further analysis at each prepulse intensity revealed that at PP4 PPI was lower in Meth-treated WT mice than in saline-treated WT mice but higher in Meth-treated HRM than in saline-treated HRM (F(1,100) = 4.97, *p* = 0.028). No differences were found at other prepulse intensities (Figure 3). Startle amplitudes were lower in female mice than in male mice (F(1,96) = 19.7, *p* < 0.001, not shown, see Supplementary Table S1) but there were no other main effects or interactions.

### 3.5. Effect of Meth Pretreatment on Sociability and Social Preference in WT and HRM

**Figure 4:**
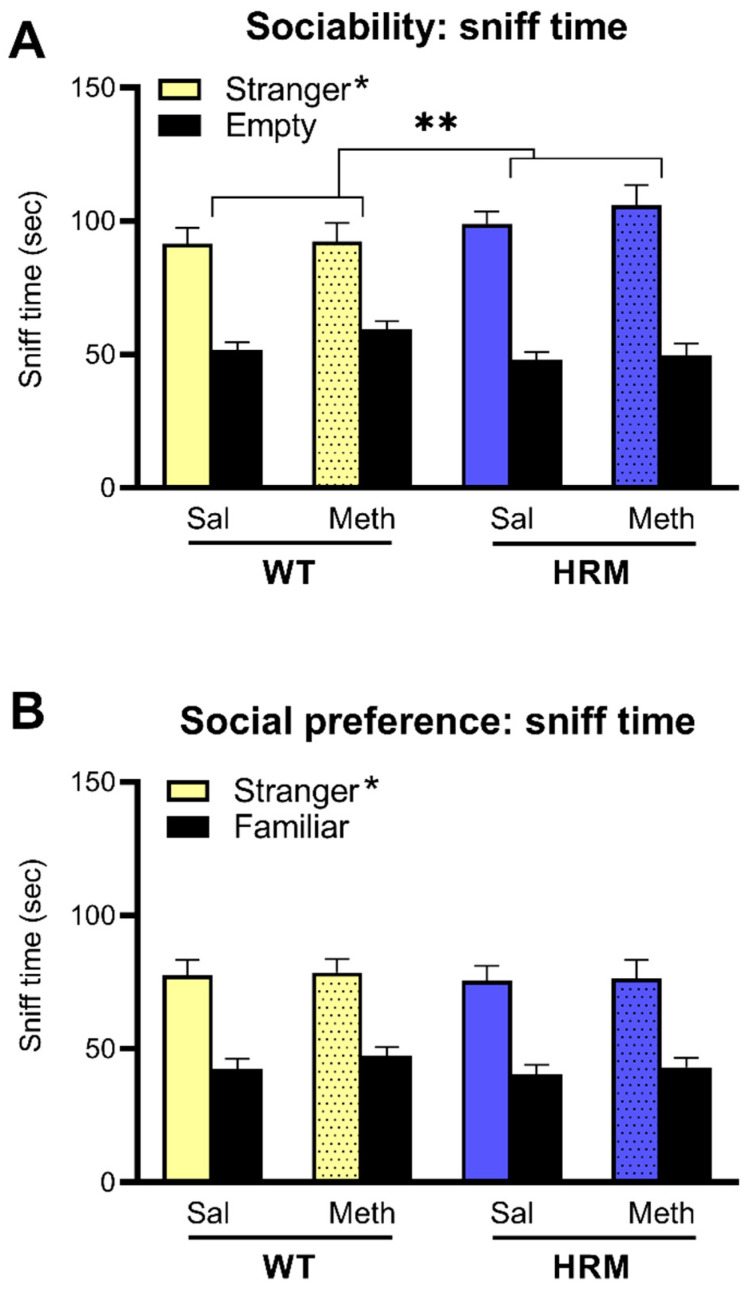
Social behaviour of WT and HRM pretreated with saline or Meth. Panel **A** shows sociability where mice showed significantly higher sniff time around the enclosure containing the stranger mouse as opposed to the empty enclosure (* *p* < 0.05). This difference was greater in HRM than in WT (** *p* < 0.05). Panel **B** shows social preference in the groups expressed as significantly higher sniff time around the enclosure containing the novel stranger compared to the familiar stranger (* *p* <0.05). There were no effects of Meth pretreatment. Data are mean ± SEM of males and females combined

Reflecting sociability, all mice showed a significant preference for the enclosure with the stranger mouse (F(1,84) = 119.1, *p* < 0.001). This difference was greater in HRM than in WT mice (Stranger × Genotype interaction; F(1,84) = 4.48, *p* = 0.037). However, there were no effects of Meth on sociability in either genotype (Figure 4A). In the social preference phase of the test, mice showed greater interaction with the novel stranger as compared to the now familiar mouse (F(1,84) = 107.0, *p* < 0.001). This was greater in male mice than in female mice (F(1,84) = 7.39, *p* = 0.008, not shown, see Supplementary Table S1) but there were no effects of genotype or Meth on social preference or any relevant statistical interactions (Figure 4B). Further details of sociability and social preference behaviour are presented in Supplementary Table S1.

## 4. Discussion

The aim of this study was to investigate the effects of chronic Meth on behaviour of HRM and WT mice, with the ultimate aim to better understand the possible role of reelin in schizophrenia. Specifically, we assessed spontaneous exploratory locomotor activity, acute Meth-induced locomotor hyperactivity, PPI and social behaviour, commonly used behavioural models of aspects of schizophrenia [33,35,40]. Chronic Meth induced the expected enhancement of the effect of an acute Meth challenge, reflecting dopaminergic sensitisation, but it had no effect on baseline PPI or sociability and social preference. In contrast to our hypotheses, HRM did not show any relevant differences to WT which would reflect a schizophrenia-like phenotype. Instead, while baseline exploratory locomotor activity was higher in HRM than WT, chronic Meth treatment reduced this activity to the level of WT. Sociability was slightly enhanced, rather than reduced, in HRM. Locomotor hyperactivity sensitisation was not significantly different between HRM and WT. Baseline PPI was not altered in HRM and no deficits in PPI or social behaviour were induced by chronic Meth pretreatment in these mice. Thus, these data do not support a role of reelin in schizophrenia, at least not in this methamphetamine sensitisation model and following heterozygous depletion.

There is considerable variability in the literature regarding schizophrenia-relevant behavioural alterations in HRM. In addition, some studies used mice with complete knockout of reelin instead of the heterozygous mutation used in the present study. For example, Matsuzaki and colleagues reported attenuated effects of Meth in homozygous reelin mutants, but not in HRM [41]. A similar observation was published by Salinger et al. [42] who found behavioural deficits in homozygous reelin mice but not HRM. We chose not to use full knockouts because it is known that these animals display marked disorganization of cortical and hippocampal layering [43,44] as well as malformation of midbrain dopamine cell groups [45]. Moreover, the reduction of reelin levels in post-mortem schizophrenia brains was reported to be approximately 50% [3], comparable to the levels in HRM [6], rather than complete depletion.

To the best of our knowledge, this is the first study to assess the effect of chronic Meth treatment in HRM. Previous studies have shown that hyperlocomotion induced by acute Meth treatment was not altered in HRM [41]. We similarly observed that acute amphetamine-induced locomotor hyperactivity was not altered in HRM [21]. Spontaneous exploratory locomotor activity measured during the one-hour habituation period prior to saline or Meth challenge, was slightly, but significantly higher in HRM compared to WT but, surprisingly, chronic Meth pretreatment reduced rather than enhanced this hyperactivity. The mechanism behind this Meth-induced reduction of exploratory locomotor activity remains unclear, particularly because in the absence of an acute drug challenge the involvement of changes in other neurotransmitter systems, such as noradrenaline or 5-HT, cannot be ruled out. It has previously been shown that the effect of an amphetamine challenge in sensitised rats was associated with enhanced dopamine release in the nucleus accumbens [46], showing the importance of an acute drug challenge. It is unlikely from our locomotor hyperactivity data that HRM showed enhanced sensitisation of subcortical dopamine release following chronic Meth treatment and, by extension, these data do not support a role of reelin in the development of psychosis.

With regards to PPI, some previous studies have shown that drug-naive adolescent and adult HRM show deficits in PPI compared to WT littermates [9,10]. However, we and others have shown similar baseline PPI in HRM compared to WT controls [21,42,47]. Similar to locomotor hyperactivity, PPI has not been studied following chronic Meth treatment in HRM. Studies in rats have shown that chronic amphetamine treatment results in disruption of PPI up to sixty days after the last injection [36]. In contrast, we have previously used a similar Meth pretreatment protocol as in the present study and showed it induced no changes in baseline PPI in WT mice [31]; the results here confirm this finding in WT and HRM. The only exception was a small increase of PPI following chronic Meth at one prepulse intensity and one inter-stimulus interval in HRM, as opposed to a small decrease of PPI in WT. It is unclear what the functional significance is of this highly selective, but opposite effect of chronic Meth pretreatment between HRM and WT. Either way, the result does not represent the reduction of PPI we expected to see [36] particularly in HRM, again not supporting a phenotype with similarity to schizophrenia, where several studies have demonstrated significant disruption of PPI [48,49,50]. It may be that any differences in PPI regulation in Meth-pretreated HRM and WT mice will only become apparent when the mice are given appropriate challenge drugs, such as acute Meth or the dopamine receptor agonist, apomorphine. We previously showed that the effect of apomorphine to reduce PPI was not different between HRM and WT but further studies are needed to address whether this is also found after Meth pretreatment.

A reduction in social behaviour represents one of the core negative symptoms described in schizophrenia and can be modelled in animals [33,38]. Preliminary studies have reported deficits of social interaction in HRM [9]. Other studies, however, observed that social sniffing, following partner, and partner climbing were not reduced in HRM compared to WT controls [47]. Using a similar three-chamber apparatus as in the present study, we previously found no changes in baseline sociability in HRM, although males had a mild social preference deficit [51]. This was not found in the present study where, instead, HRM showed a slight increase in sociability. These behavioural changes are subtle and may be explained by environmental factors such as different housing and experimental conditions [10]. We previously showed that Meth treatment, using a similar protocol as the one used here, did not alter sociability but significantly reduced social preference in WT mice [32]. In the present study, we expected chronic Meth to induce deficits in sociability and social preference particularly in HRM, however this was not found. One caveat with assessing sociability is that changes in olfactory function may affect sociability scores. In the present study we did not test or exclude for potential olfactory abnormalities. The possibility for such olfactory changes will need to be addressed in future studies.

## 5. Conclusions

In conclusion, chronic Meth treatment resulted in long-term enhancement of locomotor hyperactivity to an acute Meth challenge, reflecting sensitisation similar to that seen in schizophrenia [12,14,15,17,26]. However, this effect was not different between HRM and WT controls. Despite subtle changes in baseline exploratory activity, PPI and social behaviour, these behavioural domains were also not different between the genotypes and chronic Meth did not induce deficits in HRM reflective of a schizophrenia-like state. Overall, these data do not support a role of reelin in schizophrenia, at least not in HRM and in the methamphetamine sensitisation model. Our findings do not exclude a role of reelin in schizophrenia via other neurotransmitter pathways e.g. glutamate.

## Figures and Tables

**Table 1:** Number of animals per group per behavioural test.

Sex/Genotype	Pretreatment	Locomotor Hyperactivity	PPI	Social Behaviour
Male WT	Saline	12	12	11
	Meth	19	19	14
Male HRM	Saline	11	12	12
	Meth	14	15	11
Female WT	Saline	12	12	10
	Meth	12	12	12
Female HRM	Saline	11	11	11
	Meth	11	11	11

## Supplementary Materials

The following can be found at https://www.mdpi.com/2218-273X/10/6/940/s1: Supplementary Table S1.
