# Supplementary material for: The Effect of Chronic Methamphetamine Treatment on Schizophrenia Endophenotypes in Heterozygous Reelin Mice: Implications for Schizophrenia
Source: Biomolecules. 2020 Jun 22;10(6):940. doi: 10.3390/biom10060940 (PMC7355789; doi:10.3390/biom10060940)
Supplement: Supplementary file 1 [file biomolecules-10-00940-s001.pdf]

biomolecules

The effect of chronic methamphetamine treatment on schizophrenia endophenotypes in heterozygous Reelin mice: implications for schizophrenia

Camilla Hume, Shelley Massey and Maarten van den Buuse *

School of Psychology and Public Health, La Trobe University, Melbourne, Australia; c.hume@latrobe.edu.au (C.H.); shelley.massey.8@gmail.com (S.M.)

* Correspondence: m.vandenbuuse@latrobe.edu.au

Supplementary Table 1.

Additional relevant behaviours and data separated for males and females. For number of animals per group, see Table 1. Data are mean ± SEM.

	Saline Pretreatment				Meth Pretreatment				Significant differences?
**Behavioural parameter**	**Male WT**	**Female WT**	**Male HRM**	**Female HRM**	**Male WT**	Female WT	Male HRM	Female HRM	
**Locomotor Total Distance (cm) Moved Post-Injection - Total Arena**									
Total distance saline	7478 ± 805	6530 ± 1101	8765 ± 1571	10531 ± 2462	8220 ± 1150	5559 ± 1125	7508 ± 1149	6805 ± 1829	
Total distance Meth 1 mg/kg	6812 ± 961	6729 ± 1126	7734 ± 2525	7043 ± 1597	16434 ± 2043	14828 ± 1723	11943 ± 1666	13291 ± 2558	Acute Meth 1mg/kg vs. saline: Main effect Acute Meth F(1,94)=34.1, $p < 0.001$ Acute Meth x Meth pretreatment interaction F(1,94)=51.3, $P < 0.001$
Total distance Meth 3 mg/kg	34990 ± 3138	37910 ± 2269	35499 ± 4802	43539 ± 5562	53753 ± 3010	54419 ± 3612	49777 ± 3336	55601 ± 4604	Acute Meth 3mg/kg vs. saline: Main effect of Acute Meth F(1,95)=838.6, $P < 0.001$; Acute Meth x Meth pretreatment interaction F(1,94)=40.8, $p < 0.001$
**Locomotor Total Distance Moved (cm) Post-Injection - Inner Zone[(1)]**									
Total distance saline	1704 ± 201	1215 ± 244	2013 ± 414	2629 ± 743	1931 ± 364	1026 ± 244	1869 ± 317	1673 ± 625	
Total distance	1294	662	1375	1097	2639	1473	2131	1946	Acute Meth 1 mg/kg vs. saline:

Meth 1 mg/kg	± 248	± 146	± 547	± 255	± 405	± 402	± 338	± 498	Acute Meth x Meth pretreatment interaction $F(1,95)=21.5$, $p < 0.001$.
Total distance Meth 3 mg/kg	6733 ± 946	3373 ± 574	6792 ± 1109	4383 ± 912	8045 ± 929	5322 ± 859	9425 ± 1336	5750 ± 1044	Acute Meth 3mg/kg vs. saline: Main effect of Acute Meth $F(1,95)=131.6$, $p < 0.001$; Acute Meth x Meth pretreatment interaction $F(1,95)=8.8$, $p = 0.004$; Acute Meth x Sex interaction $F(1,95)=11.7$, $p = 0.001$; main effect of Sex $F(1,95)=17.5$, $p < 0.001$.
**PPI[(2)]**									
Average %PPI 30 msec ISI	11.5 ± 4.6	25.5 ± 3.7	22.0 ± 5.5	21.6 ± 4.8	7.7 ± 6.7	12.9 ± 5.9	16.5 ± 9.8	21.9 ± 3.1	No significant differences between groups
Average %PPI 100 msec ISI	17.6 ± 5.1	23.0 ± 2.9	24.9 ± 4.2	15.8 ± 4.0	14.0 ± 4.5	16.1 ± 4.2	20.0 ± 5.8	24.9 ± 3.9	No significant differences between groups
Average startle (units)	157 ± 29	116 ± 8	178 ± 27	130 ± 8	154 ± 11	121 ± 12	215 ± 19	115 ± 11	Main effect of Sex ($F(1,96)=19.7$, $p < 0.001$)
**Sociability: Time in Chambers (sec)[(3)]**									
Sociability: central	130 ± 8	129 ± 10	128 ± 5	134 ± 10	125 ± 12	106 ± 7	124 ± 7	108 ± 7	Main effect of Meth pretreatment $F(1,84)=5.5$, $p = 0.022$
Sociability: stranger	269 ± 10	272 ± 11	289 ± 9	276 ± 12	278 ± 18	266 ± 11	300 ± 22	297 ± 11	No significant differences between groups. Difference with empty enclosure chamber $F(1,84)=74.2$, $p < 0.001$
Sociability: empty enclosure	200 ± 10	199 ± 11	184 ± 7	190 ± 15	198 ± 13	228 ± 12	176 ± 19	196 ± 12	No significant differences between groups.
Social preference: central	130 ± 5	167 ± 11	144 ± 6	155 ± 10	143 ± 12	142 ± 8	157 ± 11	150 ± 8	No significant differences between groups
Social preference: novel stranger	266 ± 17	255 ± 10	271 ± 10	251 ± 14	265 ± 15	249 ± 15	262 ± 18	258 ± 15	No significant differences between groups. Difference with familiar stranger chamber $F(1,84)=52.7$, $p < 0.001$
Social preference: familiar stranger	204 ± 16	179 ± 11	186 ± 9	194 ± 14	192 ± 11	210 ± 16	180 ± 14	192 ± 13	No significant differences between groups
**Sociability: Frequency in Chambers[(4)]**									
Sociability: central	30.5 ± 2.9	28.9 ± 2.5	28.7 ± 2.6	26.4 ± 1.9	32.9 ± 3.3	21.9 ± 1.6	28.2 ± 2.7	23.1 ± 1.6	Main effect of Sex $F(1,84)=7.4$, $p = 0.008$, no interactions.
Sociability: stranger	14.8 ± 1.6	14.6 ± 1.3	14.3 ± 1.5	13.5 ± 1.4	17.0 ± 1.9	11.4 ± 1.0	15.0 ± 1.4	11.6 ± 1.0	Main effect of Sex $F(1,84)=6.0$, $p = 0.016$, no interactions.
Sociability: empty	15.6 ± 1.4	14.2 ± 1.4	14.1 ± 1.2	12.7 ± 0.8	15.6 ± 1.7	10.2 ± 1.0	12.8 ± 1.4	11.5 ± 1.0	Main effect of Sex $F(1,84)=6.4$, $p = 0.013$,

enclosure									no interactions.
Social preference: central	38.4 ± 2.4	35.3 ± 2.4	41.8 ± 1.9	32.2 ± 2.4	41.1 ± 3.3	30.7 ± 2.7	43.1 ± 4.2	31.5 ± 2.5	Main effect of Sex F(1,84)=18.5, $p < 0.001$, no interactions.
Social preference: novel stranger	19.7 ± 1.5	20.7 ± 1.8	21.4 ± 1.3	16.4 ± 1.4	19.9 ± 1.7	15.8 ± 1.7	21.4 ± 2.0	16.9 ± 1.4	Main effect of Sex F(1,84)=7.9, $p = 0.006$, no interactions. Difference with familiar stranger F(1,84)=5.7, $p = 0.019$.
Social preference: familiar stranger	18.5 ± 1.5	14.7 ± 1.3	20.3 ± 1.0	15.8 ± 1.4	21.0 ± 1.7	14.7 ± 1.4	21.4 ± 3.0	14.1 ± 1.3	Main effect of Sex F(1,84)=21.3, $p < 0.001$, no interactions.
**Social Preference: Sniff Time (sec)(5)**									
Sociability: stranger	90 ± 8	94 ± 9	98 ± 7	99 ± 7	95 ± 10	90 ± 10	110 ± 13	102 ± 9	No significant differences between groups. Difference with empty enclosure F(1,84)=119.1, $p < 0.001$; Stranger x Genotype interaction F(1,84)=4.5, $p = 0.037$.
Sociability: empty enclosure	52 ± 3	52 ± 5	48 ± 4	48 ± 5	56 ± 4	63 ± 5	49 ± 6	51 ± 7	No significant differences between groups
Social preference: novel stranger	91 ± 8	64 ± 6	84 ± 8	67 ± 8	85 ± 6	72 ± 9	73 ± 7	80 ± 12	Main effect of Sex F(1,92)=4.9, $p = 0.030$, no interactions. Difference with familiar stranger F(1,84)=107.0, $p < 0.001$; Stranger x Sex interaction F(1,84)=7.4, $p = 0.008$.
Social preference: familiar stranger	44 ± 5	40 ± 7	36 ± 4	46 ± 6	44 ± 4	52 ± 5	40 ± 6	46 ± 4	No significant differences between groups
**Social Preference: Sniffing Bout Frequency**									
Sociability: stranger	96.6 ± 4.8	85.9 ± 7.3	106.1± 6.2	89.5 ± 8.4	95.6 ± 8.3	91.7 ± 6.2	103.6 ± 9.2	99.7 ± 8.9	No significant differences between groups. Difference with empty enclosure F(1,84)=60.4, $p < 0.001$)
Sociability: empty enclosure	72.4 ± 6.8	66.1 ± 6.2	63.1 ± 5.0	54.9 ± 4.6	69.0 ± 4.7	71.3 ± 6.5	62.4 ± 7.7	65.5 ± 7.4	No significant differences between groups
Social preference: novel stranger	100.8± 8.2	77.4 ± 6.2	86.8 ± 7.1	69.8 ± 7.0	89.2 ± 7.3	70.6 ± 6.6	88.4 ± 8.5	81.8 ± 6.9	Main effect of Sex F(1,84)=10.0, $p = 0.002$, no interactions. Difference with familiar stranger F(1,84)=58.1, $p < 0.001$.
Social preference: familiar stranger	65.5 ± 7.5	52.2 ± 4.5	57.6 ± 7.5	52.3 ± 4.6	58.8 ± 4.9	55.2 ± 6.1	60.1 ± 6.0	56.5 ± 5.3	No significant differences between groups

Notes:

(1) Possibly reflecting higher anxiety levels in female mice than in male mice, distance travelled in the inner zone of the locomotor photocells was significantly lower in female mice than in male mice (main effect of Sex $F(1,95)=17.5$, $p < 0.001$) and the increase in locomotor activity in the inner zone after acute treatment with 3 mg/kg of Meth was similarly smaller in female mice than in male mice (acute Meth x Sex interaction $F(1,95)=11.7$, $p = 0.001$). However, sex of the animals did not interaction with the chronic effect of Meth treatment, nor with genotype. There were no sex differences in total distance moved in the total photocell arena.

(2) Average startle was higher in male mice than in female mice (main effect of Sex ($F(1,96)=19.7$, $p < 0.001$) consistent with the higher body weight of males vs. females. There were no effects of genotype or Meth treatment or any interactions.

(3) Meth pretreatment induced an increase in the animals' tendency to explore the outer chambers of the apparatus in the sociability phase of the test, resulting in significantly lower time spent in the central chamber (main effect of Meth pretreatment $F(1,84)=5.5$, $p = 0.022$). For both the sociability and social preference phase of the test, time spent in the chamber with the (novel) stranger was significantly higher than the chamber with the empty enclosure or the familiar stranger.

(4) Frequency to enter chambers was consistently lower in female mice compared to male mice (see above) but this did not interact with either genotype or Meth pretreatment.

(5) In the social preference phase, female mice showed less sniffing than male mice ($F(1,84)=107.0$, $p < 0.001$) and less preference for the novel stranger than male mice ($F(1,84)=7.4$, $p = 0.008$). These sex differences did not interact with genotype or Meth pretreatment.
